# Palmitoleic acid content and composition-based nutritional quality of commercial omega-7 oils and supplements

**DOI:** 10.3389/fnut.2026.1871452

**Published:** 2026-07-01

**Authors:** Mona Correa, Xiaoying Zhou, Dino Athanasiadis, Veronica Benites, Bryce Doherty, Lucy Edy, Christy Piamonte, Gener Eliares, Marvin Cornejo, Ting Gong, Leon Parker, Manuel Oliveira, Walter Rakistky, James Casey Lippmeier, Jessica M. Walter, Frédéric Destaillats

**Affiliations:** Checkerspot, Inc., Alameda, CA, United States

**Keywords:** algal oil, avocado oil, fish oil, macadamia oil, nutritional lipids, omega-7, palmitoleic acid, seabuckthorn oil

## Abstract

**Introduction:**

Commercial omega-7 products vary widely in fatty acid composition and chemical form, complicating direct comparison among products. This study compared commercially available omega-7 sources using palmitoleic acid (POA; 16:1 n-7) and total omega-7, calculated as POA plus cis-vaccenic acid (18:1 n-7), as composition-based indicators of omega-7 nutritional quality.

**Methods:**

Fish oil ethyl ester (FOEE) concentrates were analyzed directly, and fatty acid methyl ester (FAME) samples prepared from high-palmitoleic algal oil, seabuckthorn oils (SBO) and powder (SBOP), macadamia oils (MO), and avocado oils (AO) were analyzed by GC-FID.

**Results:**

Marked variation was observed among products. The algal oil showed the highest POA (67.3%) and total omega-7 (70.3%). The FOEE concentrates also contained high POA (54.8–57.0%), with total omega-7 only slightly higher than POA because 18:1 n-7 was very low. MO provided moderate and consistent omega-7 levels (21.5–23.0% POA; 24.8–27.0% total omega-7), whereas AO were lower (5.6–10.0% POA; 8.9–15.9% total omega-7). SBO supplements showed the greatest variability, ranging from omega-7-rich oils (25.5–35.5% POA; 32.1–46.8% total omega-7) to supplements with low or trace omega-7 (0.2–8.3% POA; 0.2–11.1% total omega-7), reflecting differences in berry/pulp, seed oil, and blended formulations.

**Discussion:**

These results demonstrate that commercial omega-7 products are not compositionally equivalent. Based on POA and total omega-7 content, algal oil showed the highest omega-7 enrichment among triglyceride-based products, FOEE concentrates were also highly enriched in a structurally distinct ethyl ester form, MO offered intermediate and consistent omega-7 levels, and SBO products exhibited substantial variability in omega-7 composition.

## 1 Introduction

Palmitoleic acid (POA; 16:1 n-7) is a monounsaturated omega-7 fatty acid that has attracted growing interest since POA was proposed to act as a lipokine linking adipose tissue to systemic metabolism (1). Among omega-7 fatty acids, POA and *cis*-vaccenic acid (18:1 n-7) are the principal isomers relevant to foods and supplements. POA has been investigated in relation to metabolic regulation, inflammation, and skin barrier biology, whereas 18:1 n-7 is less studied clinically but contributes to total omega-7 exposure and may arise from elongation of POA or dietary sources (2–4). Over the last decade, the potential health benefits of POA have been extensively reviewed, including dedicated studies focused on metabolic disease, chronic inflammatory disorders, seabuckthorn-derived lipids, and more recently skin health (2–8, 23). Nutritionally meaningful POA occurs in relatively few oils, notably seabuckthorn berry/pulp oil (SBO), macadamia oil (MO), avocado oil (AO), selected marine oils, and more recently fermentation-derived algal oils (3–9). As a result, an expanding number of dietary supplements and specialty oils are marketed under a common omega-7 claim.

However, the clinical context for POA use is not uniform across products. In human nutrition studies, macadamia nuts-containing diets and foods have generally improved total and LDL-cholesterol when they replace more saturated fat sources (10–13), although not all comparisons with oleic acid have shown favorable outcomes (14). By contrast, supplement trials with SBO or purified marine POA have yielded more heterogeneous cardiometabolic findings (15–17), whereas standardized fish-derived POA concentrates have shown promising effects on skin hydration, barrier function, and photoaging-related endpoints (18–20). A recent synthesis by Oliveira et al. places these findings within a broader skin-health framework, emphasizing that oral POA trials to date most consistently support barrier-related and photoaging-related outcomes rather than broad skin health related claims (4).

From a compositional standpoint, products marketed as omega-7 are also likely to differ substantially. SBO formulations may contain POA-rich berry/pulp oil or seed oil rich in linoleic and alpha-linolenic acids, and they may also be blended with other vegetable oils (5–8). Marine products may be offered as native oils or as ethyl ester concentrates, while newer algal oils are triglyceride-based but compositionally distinct and potentially more scalable (4, 9). Because POA is the principal omega-7 fatty acid of interest and cis-vaccenic acid (18:1 n-7) can further contribute to total omega-7 exposure, label claims alone do not necessarily provide an accurate estimate of the nutritionally relevant omega-7 fraction. This variability is not merely descriptive: it influences whether relevant POA intakes can be achieved in a practical number of softgels or in realistic serving sizes of functional foods.

**Figure 1:**
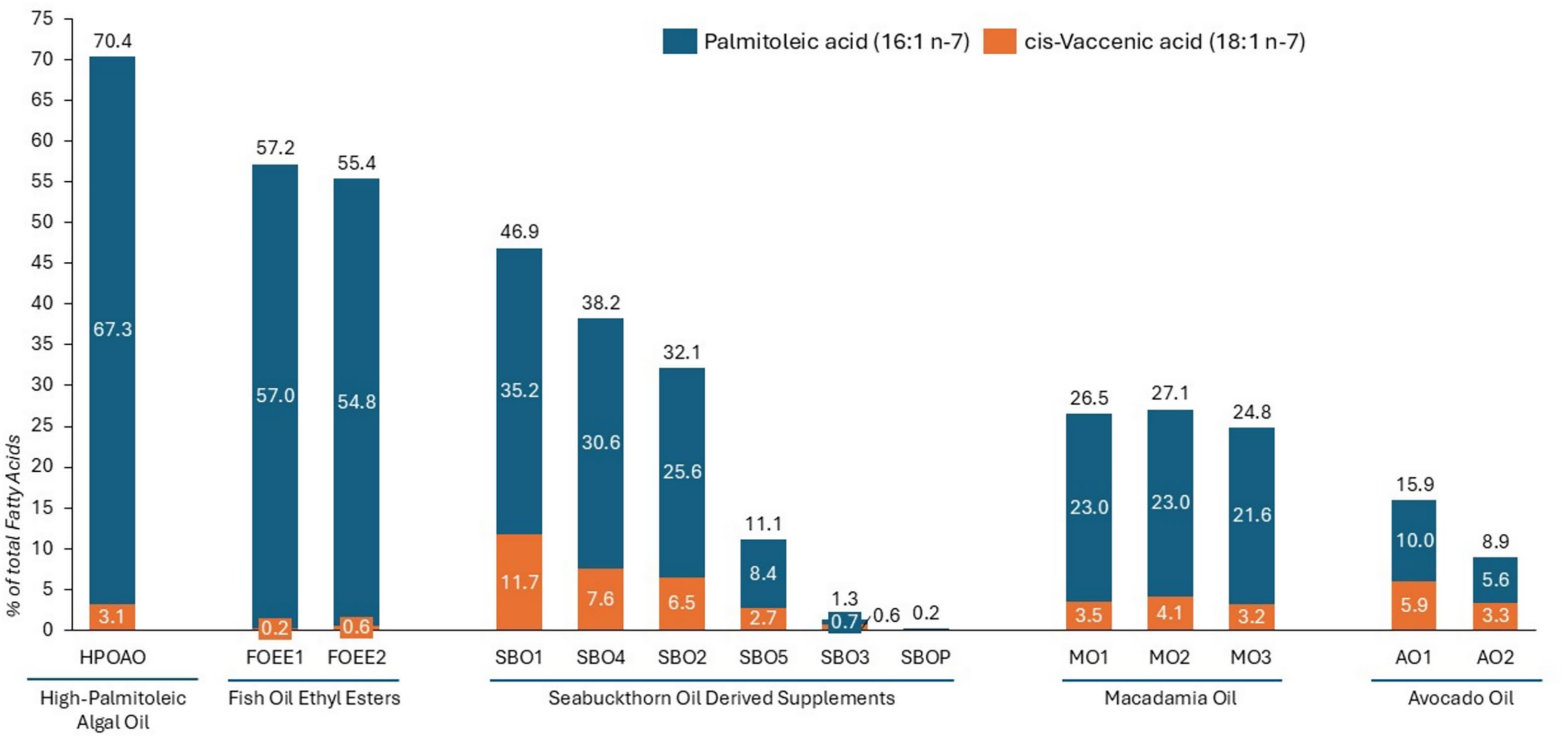
Total omega-7 fatty acids comprise palmitoleic acid (16:1 n-7, POA, blue) and *cis*-vaccenic acid (18:1 n-7, orange) across fish oil ethyl ester concentrates (FOEE), high-palmitoleic acid algal oil (HPOAO), seabuckthorn oil-based dietary supplements (SBO), macadamia oil (MO), and avocado oil (AO) samples. Values are expressed as % of total fatty acids or % of total fatty acid ethyl esters for FOEE samples.

**Figure 2:**
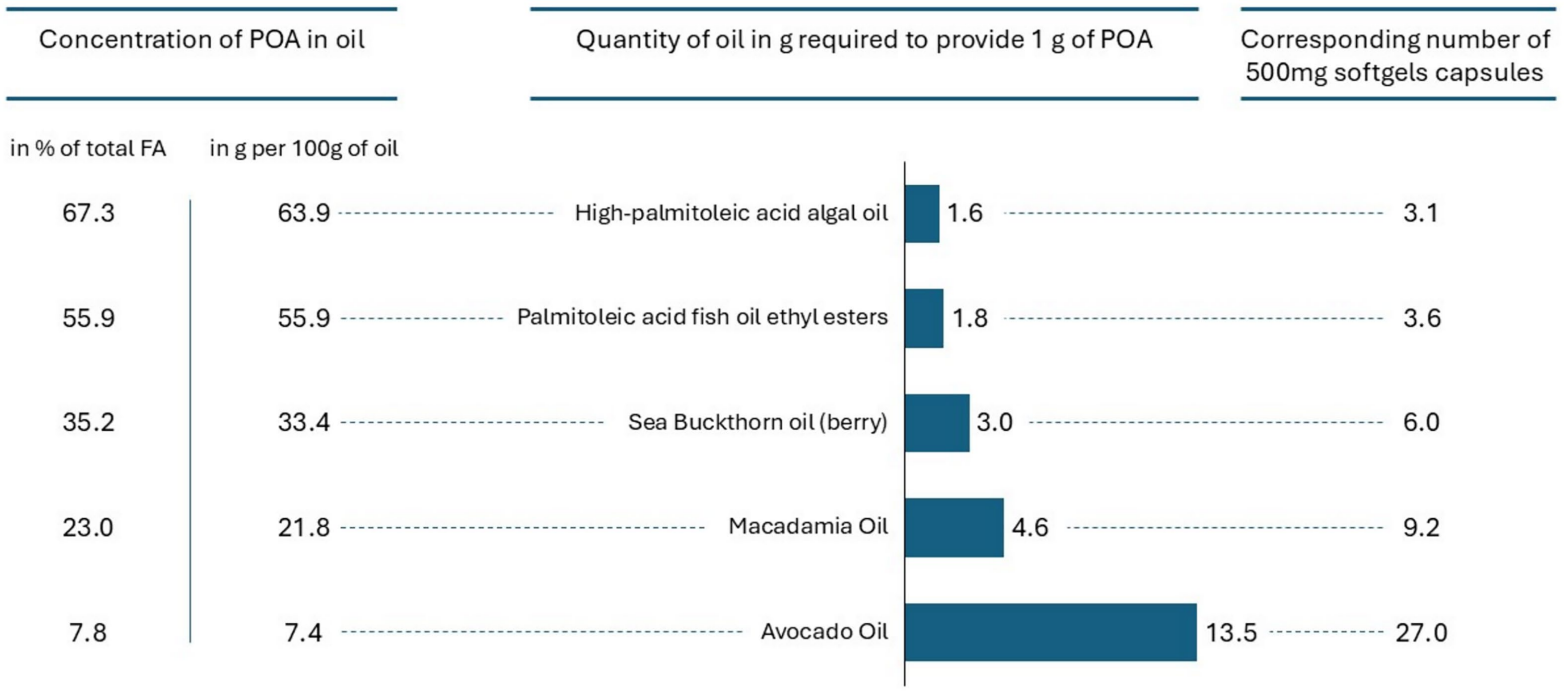
Quantity of oil required to provide 1 g of palmitoleic acid (16:1 n-7, POA) when formulating dietary supplements or functional foods using fish oil ethyl ester (FOEE) concentrate, high-palmitoleic acid algal oil (HPOAO), seabuckthorn berry oil (SBO1 sample), macadamia oil (average of MO1 and MO2), and avocado oil (average of AO1 and AO2). Corresponding equivalent numbers of 500 mg softgel capsules are provided on the right side of the figure. Estimates of the oil quantity required to deliver 1 g of palmitoleic acid (POA) were calculated from the measured POA concentration of each oil sample, expressed as grams of POA per 100 g of oil. The corresponding number of softgel capsules was projected by assuming that each capsule contains 500 mg of oil.

Therefore, the aim of the present study was to compare commercially available omega-7 products derived from fish, algae, seabuckthorn, macadamia, and avocado using GC-FID, with emphasis on POA and total omega-7 (16:1 n-7 + 18:1 n-7) as composition-based indicators of omega-7 content and quality. In this manuscript, nutritional quality refers specifically to this composition-based omega-7 level. By combining detailed fatty acid profiling (Table 1) with graphical comparison of omega-7 ranking (Figure 1) and formulation-relevant oil quantities needed to deliver POA (Figure 2), this study provides a practical framework for clinicians, formulators, and consumers to compare POA-focused dietary supplements and functional food ingredients in light of the broader literature (2–9).

**Table 1:** Fatty acid profile, average and standard deviation of duplicate analyses expressed in % of total FA, of fish oil ethyl ester (FOEE) concentrate, high-palmitoleic acid algal oil (HPOAO), seabuckthorn oil-based dietary supplements (SBO and SBOP), macadamia oils (MO), and avocado oils (AO).

Sample	FOEE1	FOEE2	HPOAO	SBO1	SBO2	SBO3	SBO4	SBO5	SBOP	MO1	MO2	MO3	AO1	AO2
14:0	0.61 ± 0.005	0.64 ± 0.000	1.49 ± 0.036	0.43 ± 0.000	0.38 ± 0.000	0.07 ± 0.000	0.18 ± 0.000	0.20 ± 0.000	2.54 ± 0.015	0.83 ± 0.000	0.83 ± 0.010	0.83 ± 0.005	0.05 ± 0.000	0.05 ± 0.000
16:0	0.92 ± 0.000	0.99 ± 0.000	6.29 ± 0.237	29.64 ± 0.015	29.23 ± 0.050	6.44 ± 0.005	24.36 ± 0.100	12.80 ± 0.050	27.84 ± 0.045	8.54 ± 0.005	8.47 ± 0.020	8.50 ± 0.015	19.92 ± 0.005	15.64 ± 0.005
16:1 n-7	56.98 ± 0.025	54.78 ± 0.070	67.26 ± 0.318	35.15 ± 0.030	25.64 ± 0.035	0.72 ± 0.000	30.55 ± 0.160	8.42 ± 0.035	0.19 ± 0.010	23.00 ± 0.005	22.97 ± 0.005	21.55 ± 0.050	9.99 ± 0.015	5.62 ± 0.005
17:1	1.27 ± 0.005	1.07 ± 0.005	0.01 ± 0.000	0.07 ± 0.000	0.07 ± 0.050	0.03 ± 0.000	0.08 ± 0.000	0.05 ± 0.000	0.03 ± 0.005	0.07 ± 0.000	0.07 ± 0.000	0.07 ± 0.000	0.09 ± 0.000	0.07 ± 0.005
18:0	0.03 ± 0.005	–	0.71 ± 0.038	0.88 ± 0.000	1.48 ± 0.000	3.41 ± 0.000	1.63 ± 0.015	2.77 ± 0.010	61.24 ± 0.110	3.17 ± 0.000	2.98 ± 0.045	3.69 ± 0.005	0.63 ± 0.000	1.315 ± 0.005
18:1 n-9	0.55 ± 0.000	1.5 ± 0.015	14.68 ± 0.129	7.74 ± 0.005	17.80 ± 0.005	20.65 ± 0.005	17.82 ± 0.110	25.87 ± 0.065	1.66 ± 0.005	51.54 ± 0.045	50.90 ± 0.510	53.24 ± 0.015	48.92 ± 0.060	54.07 ± 0.010
18:1 n-7	0.25 ± 0.000	0.65 ± 0.005	3.08 ± 0.067	11.67 ± 0.035	6.47 ± 0.005	0.61 ± 0.005	7.63 ± 0.035	2.71 ± 0.010	0.04 ± 0.000	3.45 ± 0.035	4.06 ± 0.555	3.24 ± 0.020	5.93 ± 0.000	3.26 ± 0.020
18:2 n-6	0.24 ± 0.000	0.42 ± 0.005	2.75 ± 0.050	12.12 ± 0.015	10.55 ± 0.020	66.16 ± 0.000	10.89 ± 0.055	44.65 ± 0.065	0.44 ± 0.005	1.90 ± 0.000	2.15 ± 0.085	1.71 ± 0.000	12.91 ± 0.070	18.2 ± 0.020
18:3 n-3	–	0.17 ± 0.005	0.38 ± 0.036	0.81 ± 0.000	6.60 ± 0.030	0.24 ± 0.000	5.61 ± 0.030	0.61 ± 0.000	0.14 ± 0.005	0.15 ± 0.000	0.17 ± 0.015	0.13 ± 0.000	0.79 ± 0.010	0.66 ± 0.000
20:0	–	0.03 ± 0.005	0.14 ± 0.009	0.22 ± 0.005	0.31 ± 0.000	0.24 ± 0.000	0.27 ± 0.005	0.26 ± 0.005	1.18 ± 0.005	2.70 ± 0.005	2.61 ± 0.045	2.75 ± 0.015	0.09 ± 0.005	0.21 ± 0.000
Other FA	39.4 ± 0.020	40.4 ± 0.035	3.20 ± 0.027	1.24 ± 0.005	1.45 ± 0.020	1.41 ± 0.005	0.96 ± 0.005	1.64 ± 0.050	4.66 ± 0.020	4.62 ± 0.010	4.76 ± 0.045	4.26 ± 0.045	0.66 ± 0.025	0.89 ± 0.015

Detailed information on the samples is available in Table 2. Other FA includes minor and unusual fatty acids; in FOEE samples, specific fatty acids include 16:2 n-4 and 16:3 n-4 acids as well as other minor fatty acids typically found in fish oil (see Supplementary Figure 1). The symbol “–” indicates that the corresponding fatty acid was not detected.

## 2 Materials and methods

### 2.1 Samples analyzed

A total of 14 commercial samples were procured online in the United States of America (USA) and analyzed, representing a range of dietary supplements and specialty oils enriched in or naturally containing POA as indicated by product labels (Table 2). Products were selected to cover major omega-7 categories that were available through selected online sources, rather than through a randomized or exhaustive market survey. These included dietary supplements based on fish oil ethyl esters (FOEE samples) and SBO (SBO samples), as well as specialty edible oils derived from MO (MO samples) and AO (AO samples). SBO products included berry oil, mixed seed/berry pulp oil formulations, a blend containing sunflower oil, and one powdered formulation containing magnesium stearate. High-palmitoleic acid algal oil (HPOAO) sample was obtained from Checkerspot (Alameda, CA, United States). Each product was assigned a unique sample code for identification during analysis (Table 2). Product descriptions in Table 2 are label-based. The resulting sample set is descriptive and category-oriented; it should not be interpreted as fully representative of all commercial products, brands, or lot-to-lot variability.

**Table 2:** Commercial omega-7 products analyzed in this study and their assigned sample codes.

Sample code	Product composition (based on label)	Type of product	Format
FOEE1	Palmitoleic acid fish oil ethyl esters	DS	500 mg softgel caps
FOEE2	Palmitoleic acid fish oil ethyl esters	DS	1,000 mg softgel caps
SBO1	Seabuckthorn oil (berry)	DS	500 mg softgel caps
SBO2	Seabuckthorn oil (seed/berry pulp oil blend)	DS	1,000 mg softgel caps
SBO3	Seabuckthorn oil (seed/berry pulp oil blend)	DS	1,000 mg softgel caps
SBO4	Seabuckthorn oil (seed/berry pulp oil blend)	DS	1,000 mg softgel caps
SBO5	Seabuckthorn oil (seed/berry pulp and sunflower oil blend)	DS	500 mg softgel caps
SBOP	Seabuckthorn oil, magnesium stearate	DS	1,000 mg softgel caps
HPOAO	High-palmitoleic acid algal oil	SO	Bulk oil
MO1	Macadamia oil	SO	Tin bottle
MO2	Macadamia oil	SO	Bottle
MO3	Macadamia oil	SO	Bottle
AO1	Avocado oil	SO	Tin bottle
AO2	Avocado oil	SO	Bottle

DS and SO stands for dietary supplements and specialty oil, respectively.

### 2.2 Fatty acid analysis by gas chromatography (GC-FID)

All fatty acid (FA) profiles were quantified as fatty acid methyl esters (FAMEs) following direct transesterification, as previously described (9), with the exception of the FOEE concentrates, which were analyzed directly as fatty acid ethyl esters (FAEEs). Transesterification and chromatographic analysis were performed in duplicate. Briefly, oil samples (50 mg) were submitted to transesterification reaction with a sulfuric acid-methanol solution in the presence of methyl nonadecanoate (19:0, 200 μL of a 20.0 mg/mL internal standard solution, which amounts to 4.00 mg of 19:0 added) as an internal standard for quantification of individual FAMEs. FAME and FAEE samples were analyzed using an Agilent 8,890 gas chromatograph equipped with a split/splitless inlet and a flame ionization detector (Agilent Technologies, Palo Alto, CA, United States). Chromatographic separation was performed on an Agilent DB-WAX column (30 m × 0.32 mm × 0.25 um). FAME standard mixtures 411 and 569 purchased from Nu-Chek Prep (Nu-Chek Prep Inc., Elysian, MN, United States) were used to establish retention times and response factors. The injector (split ratio 100:1) and detector temperatures were maintained at 250 °C and 270 °C, respectively. The oven programming temperature was 80 °C for 0.66 min followed by a ramp-up to 180 °C at 48 °C per min and then an increased to 275 °C at 20 °C per min and holding time of 1 min at this temperature. Hydrogen was used as carrier gas under a constant flow of 4 mL per min. Run to run reproducibility is ensured by running an internal reference standard, biomass control sample. As an example, this standard, run over 3 years and assessing 18 random runs shows a standard deviation in oil content (g/L) of 0.80 g/L (average of 49.14), oleic acid content of 0.13 area % (average of 83.60), total saturate content of 0.05 area % (average of 7.43), total monounsaturated FA content of 0.14 area % (average of 84.68) and total polyunsaturated FA content of 0.03 area % (average of 7.51). The coefficient of variation (CV%) across these five parameters was 1.62, 0.15, 0.73, 0.17 and 0.40, respectively. All peaks detected are integrated to ensure that the normalized profiling is accurate. Verification of peak identities in FOEE samples was performed by GC–MS as previously described (9).

## 3 Results

A total of 14 samples were analyzed by GC-FID, either following conversion to FAMEs or directly as FAEEs, as described in the Materials and Methods section. The samples comprised commercially available dietary supplements and specialty oils, including FOEE, HPOAO, SBO, MO and AO (Table 2). Their fatty acid compositions were compared with particular emphasis on omega-7 fatty acids, and values are summarized in Table 1 as relative percentages (% of total fatty acids).

### 3.1 Fish oil ethyl ester concentrates

The two ethyl ester samples FOEE1-2 were characterized by very high POA contents of 57.0 and 54.8%, respectively. These samples also contained the lowest levels of saturated fatty acids among all products, with total SFA of only 1.6–1.7%, and were likewise very low in PUFA (0.2–0.6%). As shown in Table 1, these samples contained approximately 40% other ethyl esters peaks. These peaks were included in the denominator used for relative percentage calculations. GC–MS characterization suggested that two major unidentified peaks (see Supplementary Figure 1) corresponded to the polyunsaturated fatty acid ethyl esters 16:2 n-4 and 16:3 n-4, which are typically present at very low concentrations in fish oil but may have been concentrated during production of the ethyl ester concentrates by short-path distillation.

### 3.2 Seabuckthorn oil supplements

The SBO products were the most heterogeneous group in the dataset, both in omega-7 content and in the balance between saturated and polyunsaturated fatty acids. Among the liquid SBO samples, SBO1 (labeled as pure berry oil) and SBO4 (labeled as a blend of seabuckthorn berry/pulp and seed oils) showed relatively high omega-7 levels, with 35.1 and 30.5% POA, respectively, and total omega-7 values of 46.8 and 38.2%. These samples also contained substantial saturated fatty acids, mainly palmitic acid (16:0), with total SFA of 31.2% for SBO1 and 26.5% for SBO4, while PUFA remained moderate at 12.9 and 16.5%. SBO2, which is labeled as a berry/pulp and seed oil blend, showed a somewhat lower omega-7 content, with 25.6% POA and 32.1% total omega-7, together with 31.4% SFA and 17.1% PUFA. In contrast, SBO3 and SBO5 appeared much less enriched in omega-7. SBO3 contained only 0.72% POA and 1.3% total omega-7 but was highly enriched in linoleic acid (18:2 n-6; 66.2%), resulting in a total PUFA content of 66.0%, the highest among all samples. SBO5, which contained sunflower oil according to the label, also showed a reduced omega-7 content (8.4% POA; 11.1% total omega-7) and a high PUFA level of 45.3%, again driven mainly by linoleic acid. These profiles are consistent with dilution of characteristic SBO (berry pulp and/or seed) by more linoleic acid-rich oils. The powdered seabuckthorn product (SBOP) was compositionally distinct from all liquid oils. It contained only trace amounts of omega-7 (0.2% POA; 0.2% total omega-7) and was dominated by saturated fatty acids, with total SFA reaching 92.8% (mainly palmitic and stearic acids), largely due to the very high level of 18:0 (61.2%). PUFA content was minimal (0.6%). This sample differed markedly from the other SBO products and from the liquid oils generally, and its composition is consistent with magnesium stearate, disclosed on the label, contributing substantially to the fatty acid profile.

### 3.3 High-palmitoleic acid algal oil

The algal oil sample (HPOAO) showed the highest omega-7 content in the present sample set, with 67.26% POA and 3.1% 18:1 n-7, corresponding to 70.3% total omega-7. In contrast to the FOEE concentrates, algal oil also contained a meaningful proportion of oleic acid (18:1 n-9; 14.7%), while remaining relatively low in SFA (8.6%) and PUFA (3.1%). Overall, this sample combined the highest omega-7 enrichment in this survey with a relatively simple fatty acid profile dominated by monounsaturated fatty acids.

### 3.4 Macadamia oil

The MO samples (MO1–MO3) showed a relatively consistent fatty acid pattern dominated by monounsaturated fatty acids. POA ranged from 21.5 to 23.0%, while 18:1 n-7 ranged from 3.4 to 4.1%, resulting in total omega-7 contents of 24.8–27.0%. These oils were also rich in oleic acid (50.9–53.2%), confirming a predominantly monounsaturated profile. In comparison with SBO, MO had more moderate SFA levels (14.9–15.8%) and very low PUFA levels (1.8–2.3%). Overall, MO represented a consistent intermediate source of omega-7 within an oleic acid-rich matrix.

### 3.5 Avocado oil

The AO samples (AO1 and AO2) contained lower omega-7 levels than MO, HPOAO, or FOEE samples. POA was 10.0% in AO1 and 5.6% in AO2, with corresponding total omega-7 values of 15.9 and 8.9%. Like MO, AO was also dominated by oleic acid (48.9–54.1%), but it contained somewhat higher levels of both SFA (17.2–20.7%) and PUFA (13.7–18.8%), particularly linoleic acid. AO therefore provided a lower and more variable omega-7 source within a broader fatty acid profile that included appreciable oleic, saturated, and polyunsaturated fractions.

Overall, the samples differed markedly in the balance between omega-7, saturated fatty acids, and polyunsaturated fatty acids. The HPOAO sample had the highest total omega-7 content, followed by the FOEE concentrates (Figure 1), which were also notable for their extremely low SFA and PUFA levels. MO contained moderate but consistent omega-7 levels and were strongly enriched in oleic acid, with low PUFA. AO samples were also oleic acid-rich but contained lower omega-7 levels. The SBO based dietary supplement products were the most variable: some samples retained the expected high omega-7 and relatively high palmitic acid profile, whereas others, especially SBO3 and SBO5, were much richer in PUFA, particularly linoleic acid, and correspondingly lower in omega-7. Finally, SBOP was an extreme outlier, being very high in saturated fatty acids and essentially devoid of omega-7 fatty acids.

## 4 Discussion

The present study shows that products marketed under a common omega-7 claim are not compositionally equivalent, with implications for composition-based POA dose estimation and product comparison. The detailed fatty acid data in Table 1 and the ranking displayed in Figure 1 show a descriptive hierarchy within the products tested: HPOAO and FOEE concentrates were the most enriched sources of POA, pure SBO berry/pulp oil occupied an intermediate position, MO provided moderate but consistent levels, and AO as well as several mixed or diluted SBO products supplied lower amounts of POA. This hierarchy is consistent with the broader literature, which identifies SBO pulp/berry oil among the richest botanical POA sources, MO as the next major plant source, avocado as a lower source, and concentrated marine oils at around 50% POA, while fermentation-derived algal oils can exceed these levels (4, 9).

From a cardiometabolic perspective, the most consistent human benefits to date have been observed with macadamia nuts based food matrices rather than with purified POA supplements. In controlled or semi-controlled interventions, macadamia-rich diets, nuts, or macadamia-enriched bread generally lowered total and LDL-cholesterol, with neutral effects on triglycerides and neutral-to-modestly favorable effects on HDL-cholesterol (10–13). These benefits appear to arise primarily when macadamia-derived monounsaturated fats displace saturated fat within the diet, which is clinically distinct from adding a POA supplement. This distinction is reinforced by Nestel et al., in which a MO-rich diet increased LDL-cholesterol similarly to palmitic acid and more than an oleic acid-rich diet (14). Accordingly, MO remains relevant for functional foods and broader dietary fat replacement, but not every POA-labeled supplement will reproduce the same cardiometabolic response. At the compositional level, the present MO samples analyzed contain 21.5–23.0% POA and about 51–53% oleic acid, which matches the profile described in the literature (3, 9).

Human data with SBO and marine POA supplements are more heterogeneous and require careful interpretation. SBO seed oil reduced blood pressure, total cholesterol, LDL-cholesterol, triglycerides, oxidized LDL, and increased antioxidant capacity in hypertensive hypercholesterolemic men (15), but the intervention product contained only about 4.9% POA and also supplied linoleic acid, *α*-linolenic acid, oleic acid, carotenoids, and vitamin E; thus, the specific contribution of POA cannot be isolated. Consistent with this caveat, a randomized crossover dose-escalation study with SBO capsules increased circulating POA isomers but did not significantly affect glucose, insulin, or lipid endpoints (16). Similarly, the 12-week trial by Bridges et al. showed that marine-derived POA capsules substantially increased circulating POA yet did not alter hs-CRP, cytokines, lipids, glucose, or insulin (17). Overall, concentrated POA supplements cannot yet be recommended as broadly effective cardiometabolic interventions, although the ongoing prediabetes trial using high-purity POA may clarify whether metabolically at-risk populations respond differently (21). Importantly, the analytical heterogeneity observed here is consistent with published source reviews showing that SBO seed oil is characteristically rich in linoleic and alpha-linolenic acids, whereas berry/pulp oil is far richer in POA (5–8).

Clinical evidence is more supportive for skin-related endpoints, especially when standardized fish-derived POA concentrates are used. In a 12-week randomized placebo-controlled trial, 500 mg/day POA from 7-MEGA improved skin hydration and reduced transepidermal water loss in middle-aged women (18). In a second 12-week trial, 1,000 mg/day POA from the same ingredient improved wrinkle parameters, hydration, elasticity, melanin index, and global photo-damage score relative to placebo (19). A third study in resistant nodulocystic acne reported favorable outcomes with a multi-component nutraceutical containing calcium palmitoleate together with calcium linoleate and magnesium phosphate (20), but the specific contribution of POA could not be isolated because the formulation contained multiple bioactive components. As summarized by Oliveira et al. (4), the current skin literature therefore provides the strongest support for oral POA in barrier function and age-associated photooxidative endpoints, while acne-related findings remain exploratory and formulation dependent. This clinical pattern is also biologically plausible because SBO pulp oil and purified POA protected UVB-irradiated fibroblasts and preserved collagen *in vitro* (22), while separate endothelial-cell experiments showed anti-inflammatory actions of POA relevant to vascular and inflammatory biology (22).

When the present compositional data are interpreted in this clinical context, pure SBO berry/pulp oil, fish oil EE concentrates, and HPOAO provided the highest POA density among the product types evaluated, but these sources differ in lipid class and should not be viewed as biologically equivalent. Among the SBO based products analyzed here, the pure berry oil retained a high POA and total omega-7 signature, whereas mixed seed/berry oils were more variable and products labeled as containing sunflower oil or formulated as powder had much lower POA (Table 1; Figure 1). This distinction is important because products marketed simply as “SBO” may differ substantially in composition and, therefore, in relevance to a POA-centered recommendation. Reviews of SBO composition consistently distinguish POA-rich pulp/berry/peel oils from seed oils, which are richer in linoleic and alpha-linolenic acids and better viewed as broader PUFA-containing botanical oils rather than omega-7-centered ingredients (5–8). Accordingly, because the existing SBO human trials used seed-oil or modified seed-oil preparations (15, 16), berry/pulp oil would be expected to deliver more POA than seed-rich preparations when the goal is specifically to increase POA intake; this statement is based on composition and should not be interpreted as evidence of superior clinical efficacy.

Figure 2 translates the compositional differences shown in Table 1 into a formulation-oriented metric: the oil mass required to deliver a target POA dose. To provide 1 g POA, only about 1.6 g of HPOAO or 1.8 g of fish oil EE is needed, compared with 3.0 g of pure SBO berry/pulp oil, 4.6 g of MO, and 13.5 g of AO. For formulation purposes, higher POA density could reduce the oil volume or number of capsules required to match a target POA intake. These formulation differences also mirror the source hierarchy described in recent reviews, in which concentrated marine oils are around 50% POA, SBO pulp/berry oils are major botanical sources, MO is intermediate, and AO contributes meaningful but clearly lower amounts (4, 9).

From an end-consumer perspective, a 500 mg softgel containing HPOAO or FOEE would provide more POA than comparable softgel capsule filled with MO or AO, assuming the measured compositions and similar fill weights. These same differences also matter for functional foods: when an ingredient is incorporated at the gram scale into beverages, bars, spreads, dairy alternatives, or other lipid-containing foods, higher-POA oils can reach meaningful daily POA exposures without excessive fat loading. This is especially relevant for SBO based product development, because berry pulp-, seed-, and blended ingredients have all been proposed for food applications but will deliver very different amounts of POA (6). From a dose-density standpoint, fish EE is well suited to softgel supplements when high, standardized POA dosing is desired, whereas pure SBO berry oil may suit botanical-focused products but generally requires larger unit fills or multi-capsule daily regimens. MO is better positioned for whole-food or formulated-food contexts that capitalize on its broader MUFA profile and clinical replacement data (10–14), while AO appears less efficient when the formulation target is POA content per se (9).

Among triglyceride-based products, HPOAO had the highest POA and total omega-7 content in the present survey while retaining a predominantly monounsaturated fatty acid profile. Together with recent reviews identifying fermentation-derived oils as emerging POA sources, these data support further composition-based evaluation of algal oil as a high-POA triglyceride ingredient (4, 9). Additional work is needed to assess oxidative stability, contaminants, minor bioactives, bioavailability, sensory and functional food performance. Finally, the present survey was limited to a small and uneven set of commercially available products, and was not designed to estimate the full breadth of market variability or lot-to-lot reproducibility. The results should therefore be interpreted as a descriptive comparison of selected products and categories rather than as market-wide prevalence data.

## 5 Conclusion

In conclusion, commercial omega-7 supplements differ markedly in both the amount and the form of omega-7 fatty acids they provide and should not be considered nutritionally equivalent on the basis of labels alone. Using POA and total omega-7 as composition-based indicators, HPOAO and FOEE concentrates showed the greatest enrichment within the sample set, whereas SBO supplements ranged from POA-rich berry oil to seed-rich, blended, sunflower-diluted, or powdered formulations with little meaningful omega-7. MO offered moderate but consistent omega-7 levels within an oleic acid-rich matrix, while AO provided lower and more variable amounts. These findings highlight the need for direct compositional verification when selecting omega-7 dietary supplements or functional food ingredients, since product labels alone do not reliably predict POA delivery. From a formulation standpoint, pure SBO berry/pulp oil, standardized fish oil EE concentrates, and HPOAO were the most concentrated POA sources among the products evaluated, with HPOAO showing the highest POA level among triglyceride-based sources tested. Future studies should compare compositionally verified products, standardize the daily POA dose and chemical form, and include assessments of stability, formulation performance, and bioavailability.

## Data Availability

The original contributions presented in the study are included in the article/Supplementary material, further inquiries can be directed to the corresponding author.
