# Supplementary material for: Palmitoleic acid content and composition-based nutritional quality of commercial omega-7 oils and supplements
Source: Front Nutr. 2026 Jul 1;13:1871452. doi: 10.3389/fnut.2026.1871452 (PMC13371309; doi:10.3389/fnut.2026.1871452)

Supplementary Figure 1

GC-MS chromatogram showing the presence of 16:2 n-4 and 16:3 n-4 ethyl esters in fish oil ethyl ester concentrates analyzed in the presence study

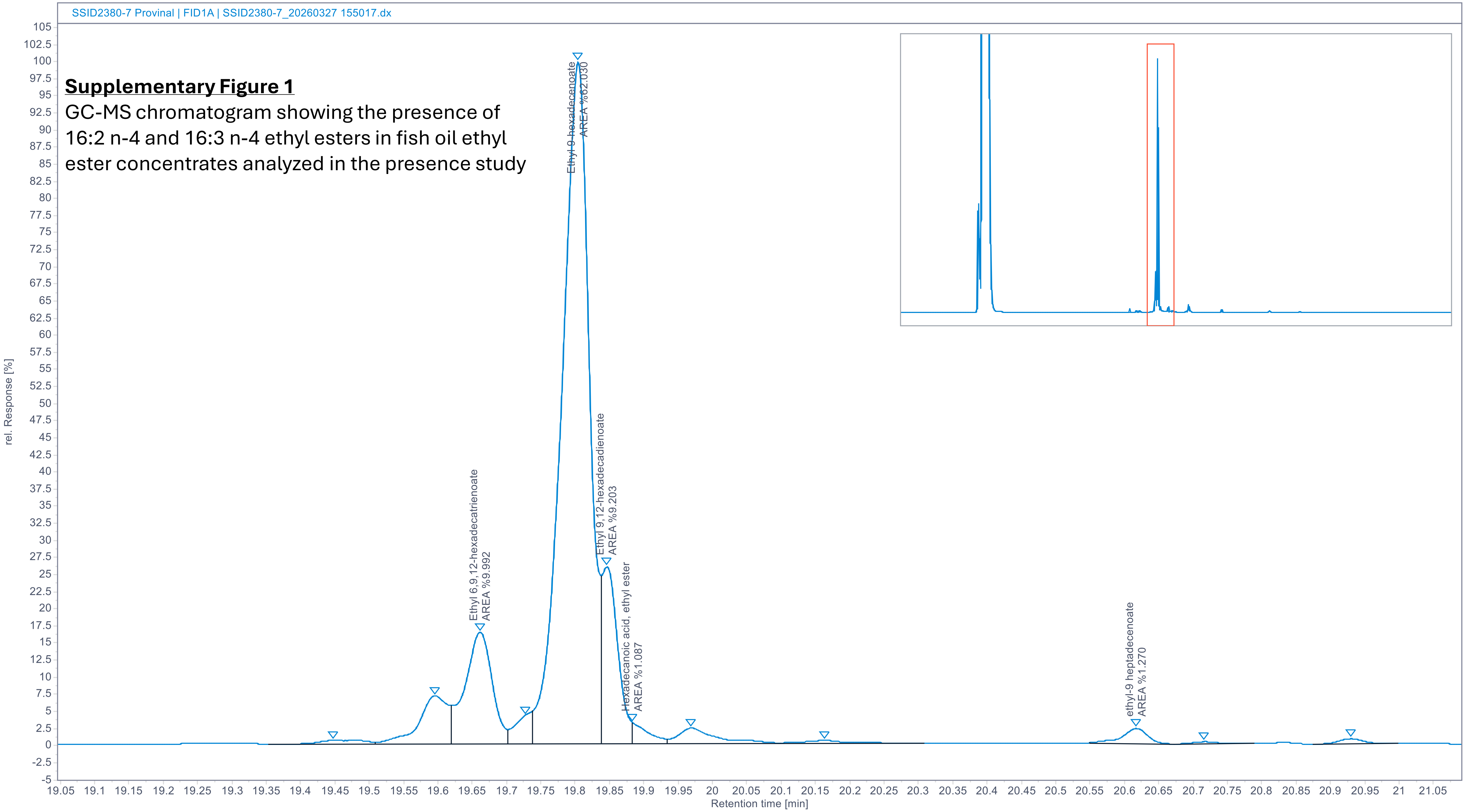

Supplement: Supplementary file 1 [file Image_1.pdf]
